# A macroscopic and stereological imaging dataset of *Pleuronectes platessa* ovaries

**DOI:** 10.1038/s41597-020-0505-8

**Published:** 2020-05-29

**Authors:** Carine Sauger, Jérôme Quinquis, Kristell Kellner, Clothilde Heude-Berthelin, Mélanie Lepoittevin, Nicolas Elie, Laurent Dubroca

**Affiliations:** 1 Institut Français de Recherche pour l’Exploitation de la Mer, IFREMER, Laboratoire Ressources Halieutiques de Port-en-Bessin, 14520 Port-en-Bessin-Huppain, France; 2 Université de Caen Normandie, Biologie des Organismes et Ecosystèmes Aquatiques (BOREA) MNHN, Sorbonne Université, UCN, CNRS-7208, IRD, UA, team EMERGE, Esplanade de la Paix, 14032 Caen, France; 3 Normandie Université, 14400 Caen, France; 4 Normandie Université, UNICAEN, SF 4206 ICORE, CMABIO3, 14000 Caen, France

**Keywords:** Cellular imaging, Urogenital models

## Abstract

The North Sea plaice, *Pleuronectes platessa* (Linnaeus, 1758), is a commonly studied commercial flatfish with poorly known ovarian histology. The following dataset is a collection of female plaice gonad images and their corresponding histological slides, collected during a complete season of the plaice’s reproduction cycle. Stereology was used to determine the percentage of different structures found throughout the ovaries. Inter-agent calibrations were accomplished in order to harmonize the stereological readings, and were based on a comprehensive reading protocol and histological lexicon that were specifically written for the plaice’s ovaries. The distribution and homogeneity of the different cell types found throughout the ovaries were also evaluated. This dataset can be used to automate the stereological reading process (through statistical learning methods for example) or to objectively determine the plaice’s maturity phase, and link that information to either macroscopic measurements or through image analysis of the full ovaries.

## Abstract (summary)

**Table:** 

Measurement(s)	female gonad • ovary morphology trait • cell type
Technology Type(s)	photography • stereology • histological assay
Factor Type(s)	phase of reproductive cycle
Sample Characteristic - Organism	Pleuronectes platessa
Sample Characteristic - Environment	sea

Machine-accessible metadata file describing the reported data: 10.6084/m9.figshare.12296054

## Background & Summary

In stock assessments, the reproductive capacity of a commercial fish species is a key parameter for fisheries management plan. This reproductive capacity, or the capacity of a fish population to produce viable eggs and larvaes^1^, is usually estimated through the computation of the fish length at which 50% of the fish population has reached sexual maturity (L50)^2^. Being able to accurately determine the maturity phase of a fish is thus of paramount importance^1,3^. Unfortunately, these methods are very subjective, with the use of criteria such as the size, color or texture of the gonads, to estimate sexual maturity of commercial species^4,5^. Moreover, the maturity cycle of certain fish species is poorly known^6^, the determination of maturity phases can show great variability between assessing operators^7,8^, there are numerous terminologies to describe the ichthyological reproductive system^4,5,9^, and maturity scales are in constant evolution and differ from one institution to another^5^. This led the International Council for the Exploration of the Sea (ICES) to work on harmonizing the definitions, terminologies and practices used to determine these different maturity phases^9^.

This study has been set under project MATO (“MATurité Objective des poissons par l’histologie quantitative” - Objective fish maturity using quantitative histology), carried out by the Institut Français de Recherche pour l’Exploitation de la Mer (IFREMER). This project aimed to bring knowledge on the ovarian histology of the North Sea plaice (*Pleuronectes platessa*) in order to correlate histological maturity with macroscopic parameters like the size, weight and age of the fish, as well as the size, color and texture of the ovaries. Moreover, in order to harmonize data collection and terminologies, the terminology used by Brown-Peterson and al^4^, and the maturity scale of the ICES^5,9^, were used. *Pleuronectes platessa* was chosen as an easy to access and important commercial fish species, with the aim to update the outdated knowledge on the histological structures of this species’ ovaries^10–13^.

**Fig. 1:**
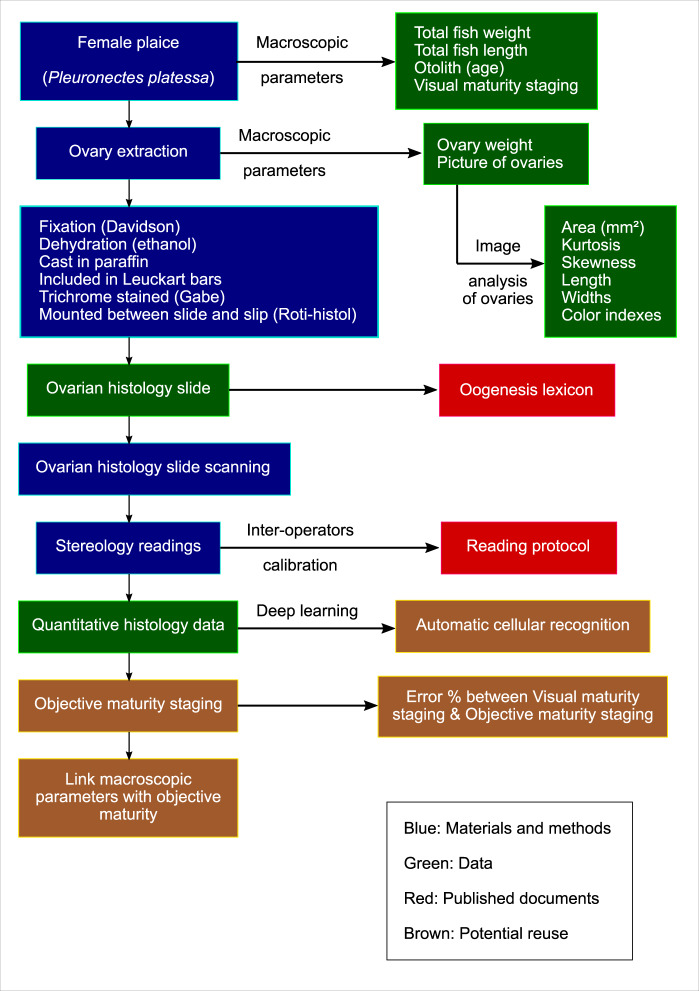
Schematic overview of the data acquisition process for *Pleuronectes platessa* macroscopic parameters, ovaries and ovarian histological slides. In blue are the materials and methods used for data collection, in green is the raw data obtained throughout the study, in red are the documents that have been published as a result of the data collection process, as well as to allow reproducibility of the slide readings, in brown are the potential use and reuse of the database.

During this study (Fig. 1), each plaice was measured (weight and size), their otoliths were collected for age estimations and the ovaries were extracted to be photographed, weighed, put into a Davidson solution before being cast into paraffin, trichrome stained (Prenant-Gabe^14^), and mounted between slide and slip. Each slides were then scanned and stereology was used to quantify each structure found throughout the ovary.

The dataset presented here is composed of:

- 151 pictures of both ovaries for the 151 fish sampled during this study;
- 226 histological slides scanned, with 151 median ventral (V2) ovarian sections and an additional 75 ovarian sections from the anterior ventral (V1), posterior ventral (V3), anterior dorsal (D1), median dorsal (D2), and posterior dorsal (D3) positions;
- Data frames with the reading results for the 226 histological slides;
- Data frame with the general data and macroscopic measurements for all 151 fish.

The fish analyzed were all females, with a size range going from 15 cm to 36 cm, weights ranging from 34 grams to 523 grams, and ages spanning from 1 year old up to 5 years old.

This dataset completes, updates and enriches the existing knowledge on plaice oogenesis, and allows a comparison between macroscopic and microscopic parameters for each fish. Using an objective histological method to determine the sexual maturity phase in *Pleuronectes platessa* is time consuming but yields better results compared to the visual method. Moreover, with this database, less time consuming methods, such as image analysis and statistical learning for the recognition of cellular structures, can be put in place. This dataset can also be used as a means to calibration between stereology readings, as well as defining objectively the sexual maturity of each individual using histology.

## Methods

### Sampling

All 151 individuals were fished by bottom trawling in the English Channel (ICES division VIId), during 10 different sampling events from January 2017 to August 2019 (Table 1) so as to gather data from fish at different phases of the reproductive cycle^15,16^. The sampling method took into consideration all captured female plaice, regardless of the size. During the data collection in August, only female plaice of 20 cm and under were dissected in order to complete previous missing data for the sexually immature (SI) state.

**Table 1:** Table with the number of fish caught at different months during the study, from 2017 until 2019.

Month	Year	Number of fish sampled
January	2017	5
December	2017	5
March	2018	10
June	2018	12
November	2018	14
December	2018	10
January	2019	24
February	2019	23
March	2019	30
August	2019	18

Each fish was measured (total length with an accuracy of less than 1 cm), weighted (ungutted weight with an accuracy of less than 1 g) and aged through otolithometry. An experienced operator estimated each female’s sexual maturity through the observation of the ovaries, following the maturity staging grids of the ICES^9^. Both ovaries were then extracted and photographed.

**Fig. 2:**
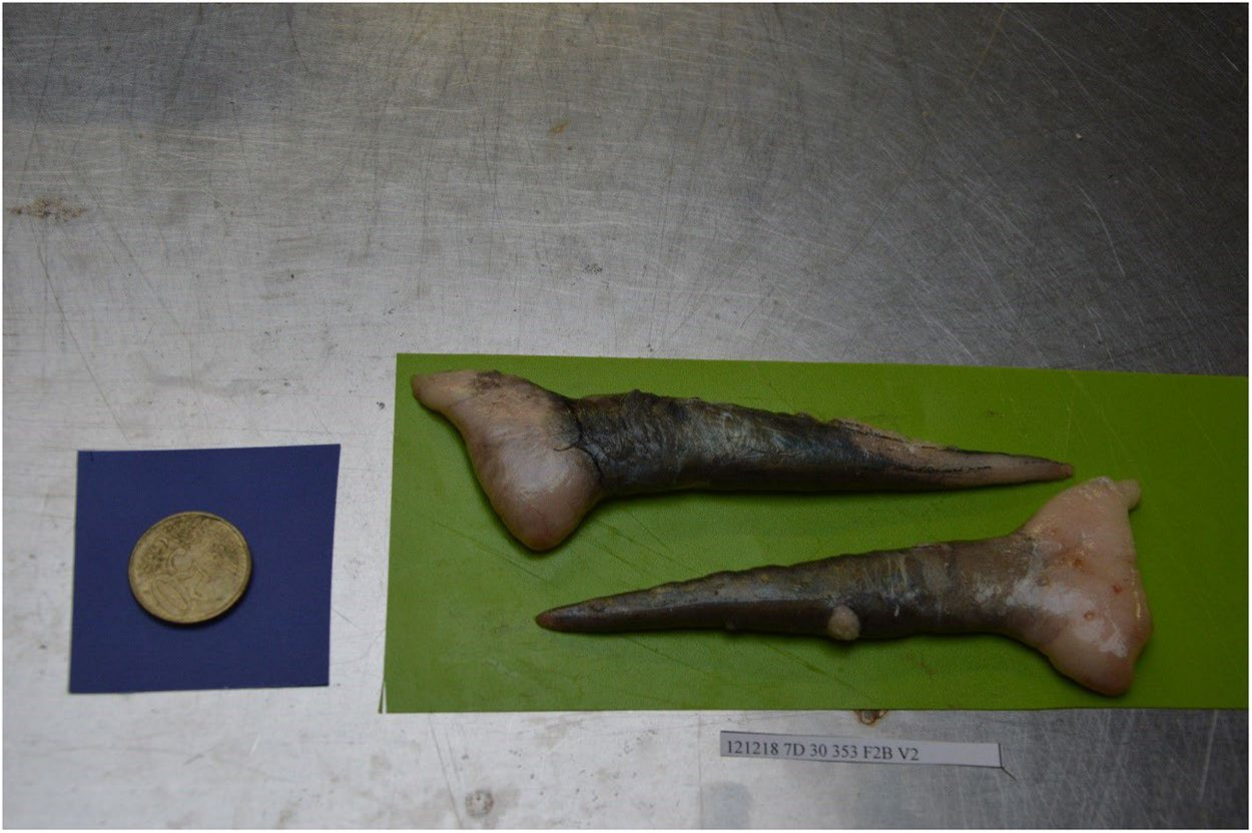
Set up for the photography of the whole gonads. Ventral (bottom) and dorsal (top) plaice ovary on a green background, with the fish’s identification number on a tag in the lower part of the picture, and a 0.50€ coin on a blue background for size calibration.

These pictures (Fig. 2) were standardized by being taken by the same operator, with the same camera (Nixon D3200), and in the same workroom so as to minimize the variations from the shot angle and lighting. The ovaries were positioned onto a green background, next to a 0.50€ coin on a blue background that served as a fixed size marker. The identification tag of the sampled fish appeared under the ovaries. The picture was named after the fish’s identification tag. Each fish’s identification tag was composed of the following data: specie’s code, date of sampling, sampling zone, total length of fish, ungutted weight of fish, sex, visually estimated maturity phase.

The Image J software (v. 1.50J) was used to analyze the pictures and calculate the macroscopic parameters of each ovary: surface (mm²), length (mm), width (mm), width at mid-length (mm), mean color value of the different hues found on the ovary, the standard deviation of the mean color value, and the modal value (the most frequently occurring color value within the selected ovary).

### Mounting between slide and slip

**Fig. 3:**
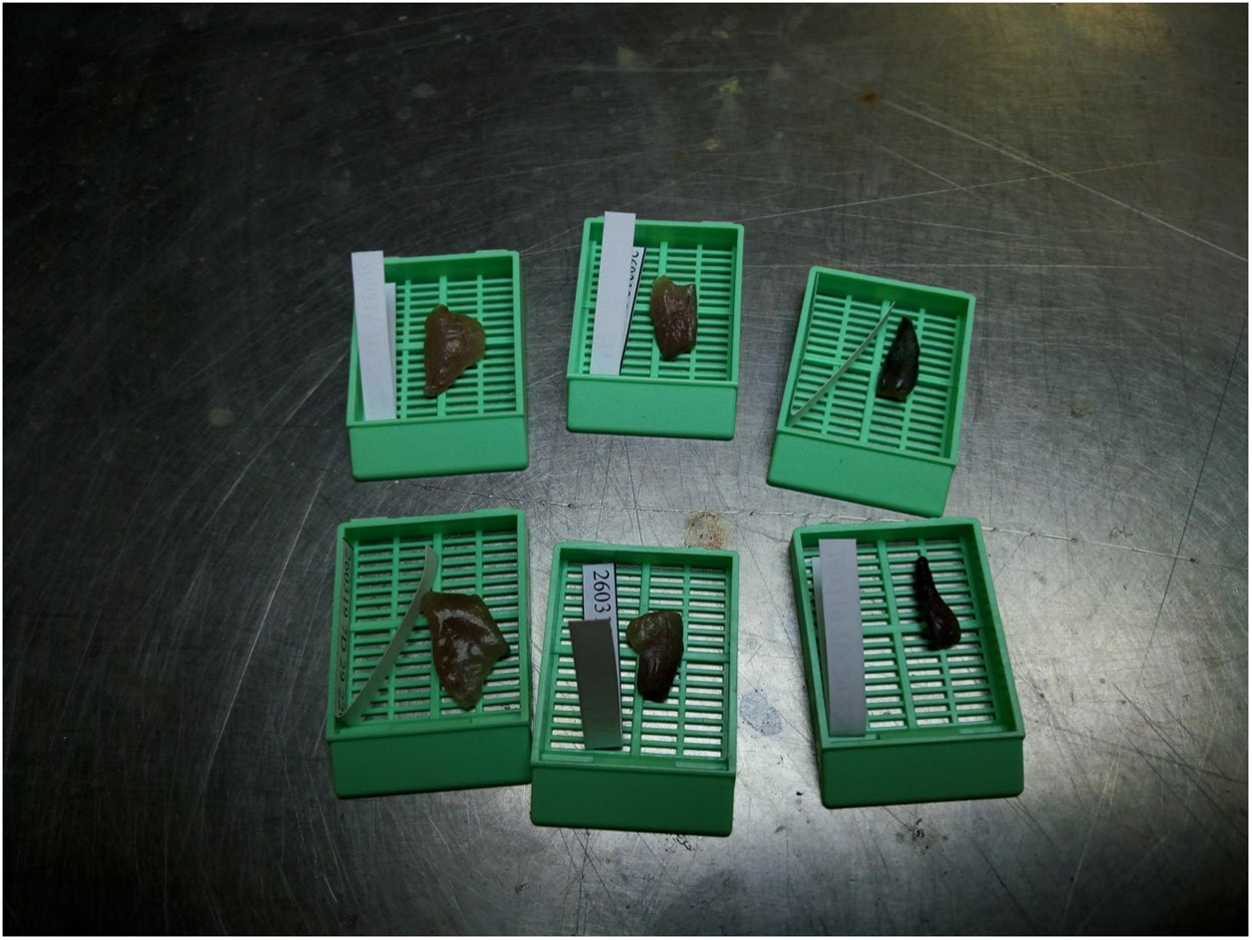
Tissue processing embedding cassettes with the 6 ovary sections from one individual, and their unique nametag. Top from left to right: section D1, D2, D3. Bottom from left to right: section V1, V2, V3.

Both ovaries were placed into separate tissue processing embedding cassettes with their respective nametag. For the individuals with ovaries of 3 centimeters and over, the dorsal ovary (coded D) and ventral ovary (coded V) were cut into 3 sections of 1 cm. These sections were located in the anterior (coded 1), median (coded 2), and posterior (coded 3) area of each ovary. The 6 samples were then placed into 6 separate cassettes (Fig. 3) with a unique nametag that included the fish’s identification tag followed by the section position (V1, V2, V3, D1, D2 or D3).

The tissue processing embedding cassettes were placed into a Davidson solution, for tissue fixation, for a period of 12 to 24 hours at a temperature of 4 °C. For the Davidson solution, 400 ml of glycerol, 800 ml of formol (37%), 1200 ml of ethanol (95%) and 1200 ml of filtered marine water were added in that order. This solution was kept at 4 °C, and 360 ml of concentrated acetic acid (10%) was added before use. The samples were then trimmed at the edges so as to get a straight rim, before being placed into an automate (Leica TP1020) for dehydration. The dehydration process lasted 48 hours with a succession of different baths: 1 hour in ethanol 70%, 3 hours in ethanol 70%, 3 hours in ethanol 95%, 4 hours in ethanol 95%, 3 hours in ethanol 100%, 4 hours in ethanol 100%, 6 hours in ethanol 100%, 2 hours in butanol 100%, 4 hours in butanol 100%, 6 hours in butanol 100%. Still in the automate, the samples were immersed into a first liquid paraffin bath (60 °C) for 4 hours before being immersed into a second paraffin bath for 8 hours.

The samples embedded in paraffin were cut into sections of 5 microns thick, using a microtome (HM330). Three consecutive sections were placed onto a single slide. Each slide was then deparaffinized, rehydrated and stained in Prenant-Gabe’s Trichrome^14^. Finally, the sections were mounted with Roti-Histokitt.

### Quantitative histology

The slides were digitized using a histology slide scanner Aperio CS, running under the Scan Scope Console software (v.10.2.0.2352, Leica Biosystems), with a magnification of 20x (numerical aperture 0.75). The scans were then analyzed using the Aperio software (v12.1.0.5029)^17^. The counting of cellular structure was done with the use of a stereological analysis based on Glagolev’s method^18^, an assumption-based stereological method that uses a grid of points to estimate the different structures’ areas on the total amount of points sampled.

**Fig. 4:**
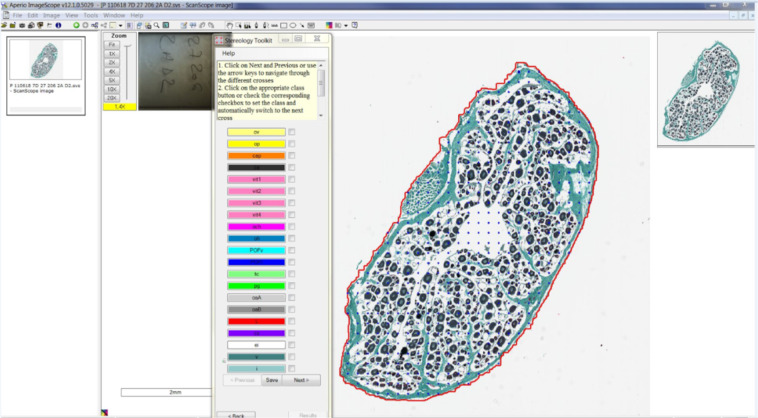
Aperio interface showing an ovarian histological section outlined by a red line that delimits the sampling area. The blue crosses are the 500 to 600 sampling points, and the Stereology Toolkit window shows the different cellular structures that can be found throughout the ovary.

Through the Aperio interface (Fig. 4), a sampling grid overlaid the scanned ovary slide. The generated sampling grid outlines the ovary, sketching a line along the outer ovarian wall, so as to reduce the sampling area to just the ovarian section. This sampling grid was composed of 500 to 600 sampling points equidistant from one another, and covering the entire sampling area. The fact that the 500 to 600 points are always equidistant from one another, and scales with the sampling area, assures the same sampling effort for every slide, no matter the surface or the shape of the outlined ovary, while covering a maximum of the sampling area. The position of the first cross is randomly generated, making the grid of sampling points unique each time a new one is created. This is important since it means that the sampling is random, but also that if multiple grids are generated for the same slide, it is important to use the same grid if we wish to compare the results of multiple readers. Finally, a 500 to 600 sampling point grid was used based on Gundersen’s rule^19^ that states that a biological compartment must have at least 150 points so as to obtain a relative uncertainty, between 3% and 10%, on the estimation of its volume’s fraction. Having more than 5 structures at all times for each slides, the 500 to 600 points are not enough to abide by that rule, but that number of sampling points still allows for an accurate count of the fraction that each structure occupies within the histological slide, without having so many points that it would make the reading excruciatingly long, or make the sampling effort amount to manually counting each cell one by one.

For each sampling point, a single cellular structure was assigned from the 20 different cellular structures that could be found throughout the ovary. These 20 structures were identified, discussed and then clearly described by experienced oogenesis specialists (KK and CHB) in a lexicon^20^. Once each sampling point was assigned to a structure, the percentage of times that a structure was counted on a single slide was calculated:

$$\begin{array}{l}Percentage\,\left( \% \right)\,of\,times\,the\,structure\,was\,counted\\ =\,\left(100/Total\,number\,of\,sampling\,points\right)\,* \,number\,of\,times\,the\,structure\,was\,counted\end{array}$$

A total of 226 histological slides were read with:

- 90 slides were read to check the cellular homogeneity between and within the gonads of each individual. These slides are the ventral anterior (V1), ventral median (V2), ventral posterior (V3), dorsal anterior (D1), dorsal median (D2) and dorsal posterior (D3) ovarian sections of 15 female plaice;
- 151 slides of the median section of the ventral ovary (V2) were read for maturity staging;
- Amongst these 151 slides, 20 slides of the median section of the ventral ovary (V2) were used for the agent calibration exercise.

## Data Records

The images and datasets generated during the current study are available in the Zenodo repository^21^, [10.5281/zenodo.3745640].

This dataset contains macroscopic (Full_Ovaries_Data.zip) and histological images (Stereology_Readings_Data.zip) of the ovaries of 151 European plaice (female, *Pleuronectes platessa*), as well as three data frames that are: the macroscopic parameters collected for each fish (Macros.csv), the stereological reading results of the calibration exercise from the 3 operators (Interagent.csv), and the stereological reading results for all 226 histological slides read throughout this study (Stereology.csv).

Images:

- Full_Ovaries_Data.zip: archive in zip format of 151 pictures (.JPG; 8Mo-9Mo; sRGB; 6016 × 4000 pixels) of both ovaries from 151 female plaice dissected during this study.
- Stereology_Readings_Data.zip: archive in zip format of two directories containing the following images:

- Interagent_Calibration: The pictures (.svs: Aperio single-file pyramidal tiled TIFF, with non-standard metadata and compression) are of the 20 histological slides used for the stereological count during the agent calibration exercise
- Ovary_Slides: The pictures (Aperio single-file pyramidal tiled TIFF, with non-standard metadata and compression) in this dataset are of the 226 histological slides read during this study

Data frames:

- Intergaent_read_me.txt: a text file (.txt) listing the acronyms used in the Interagent.csv file, as well as their meaning.
- Interagent.csv: a text data file (.csv) with the output of two stereological readings, done by three agents for 15 slides, and by two agents for 20 slides. The information contained in this table is as follows:

- agent: code id for the three agents that did the calibration exercise (A, B and C)
- num_fish: fish number for this study. Here we have 20 different fish
- fish_id: identification number of the fish. This id number is identical to the name given to the pictures of the full ovaries (Full_Ovaries_Data)
- scan_id: identification number of the digitized histological slide that was used for the stereological count (Stereology_Readings_Data/Interagent_Calibration)
- total_points: total number of identified structures for the stereological sampling grid of a slide
- cell-type: abbreviation of the structure identified (lexicon available here: https://archimer.ifremer.fr/doc/00501/61235/). In this study, we have 20 different structures
- hit_points: number of time a structure has been counted on a single slide
- Fract_estim: percentage (%) of times a structure was counted on a single slide = *(100*/total_point) * hit_points
- reading: reading number. In this study, we have two readings, the first (1) and the second (2)

- Macros_read_me.txt: a text file (.txt) listing the acronyms used in the Macros.csv file, as well as their meaning.
- Macros.csv: a text data file (.csv) containing macroscopic parameters measurements for all 151 fish that have been used during this study. The information contained in this table is as follows:

- num_fish: fish number for this study. Here we have 151 different female fish
- fish_id: identification number of the fish. This id number is identical to the name given to the pictures of the full ovaries (Full_Ovaries_Data)
- gon_pos: gonad position, with D being the dorsal gonad of the individual, and V being the ventral gonad
- date: the date the fish was caught (dd/mm/yyy)
- L_fish: total length of the fish (cm)
- W_fish: total weight of the fish (g)
- mat_estim: visually estimated maturity, after observation of the fish’s gonad with the naked eye, following the WKMATCH^9^ scale
- age: estimated age (in years) of the fish, after analysis of the fish’s otolith. The IFREMER laboratory executed this analysis in Boulogne-sur-Mer (FRANCE)
- W_gon: gonad weight (g)
- Kurtosis*: kurtosis parameter
- Skewness*: skewness coefficient
- gon_area*: gonad area (mm²)
- L_gon*: gonad length (mm)
- width_gon*: maximum gonad width (mm)
- width_mid_L_gon*: width at mid-length of the gonad (mm)
- mean_col_index*: the mean color value of the different hues found on the ovary
- std_dev*: standard deviation of the mean_col_index
- modal*: modal value or the most frequently occurring color value within the selected ovary

*: values determined after image analysis of the Full_Ovaries_Data images with the ImageJ software (v. 1.50 J)

- Stereology_read_me.txt: a text file (.txt) listing the acronyms used in the Stereology.csv file, as well as their meaning.
- Stereology.csv: a text data file (.csv) of the stereology count results of 226 slides read during this study. Among these slides, 90 were read to test the homogeneity distribution of different cell types found throughout each ovary, and 151 median histological slides of the ventral ovary were also read. The information contained in this table is as follows:

- agent: code id for the 3 agents that did the calibration exercise (A, B and C)
- num_fish: fish number for this study. Here we have a total of 151 fish
- fish_id: identification number of the fish. This id number is identical to the name given to the pictures of the full ovaries (Full_Ovaries_Data)
- scan_id: identification number of the digitized histological slide that was used for the stereological count (Stereology_Readings_Data)
- reading: reading data to test the homogeneity of cell distributions throughout the ovaries (homogeneity) or reading data of all the slides (median)
- cell-type: abbreviation of the structure identified (lexicon available here: https://archimer.ifremer.fr/doc/00501/61235/). In this study, we have 20 different structures
- point_id: identification number of the point inside the stereological sampling grid placed over the ovarian histology slide
- coord_x: x coordinate of the sampling point
- coord_y: y coordinate of the sampling point

## Technical Validation

### Inter-agent calibration and reading protocol set up

To quantify the reading disagreements between operators, the differences in cellular structure identification between several agents was assessed. A total of 15 slides were read by 3 different agents. The 15 slides are median sections of the ventral ovary (V2) of 15 different fish, and were randomly picked out from the slides at our disposal at the time of this study. For every slide, a reading error index was established for each cellular structure by calculating the difference in percentage between the maximum and the minimum counting value of each structure identified on the slide. To identify cell structures that present problems for reader identification, a threshold of 3% was set by taking the quantile at 90% of the distribution of the percentage reading index. This choice reflects a compromise between the quality of the readings (the low reading error percentage of 3%) and the recognition of significant identification problems (the selection of the 10% most error-prone structures with a reading error index higher than 3%). Consequently, for all of the structures that showed a reading error index of more than 3%, the slides were reviewed and each reader explained why they chose their respective structures for each sampling point. These results from the first reading exercise allowed the adjustment and improvement of the reading protocol^22^, as well as setting an error limit of 3% for the reading error index of each structure.

During the second reading exercise, the same 15 slides and sampling grids were read again by the same 3 operators. The results were then analyzed through the reading error index of each structure, as well as the estimation of the percentage agreement between readers^23^, and Fleiss’s kappa^24,25^. These last two inter-rater reliability indexes are statistical indexes based on the degree of agreement between readers for the classification of objects or individuals^23–25^.

### Cellular homogeneity inter and intra-gonad

To assess the cellular homogeneity inside the gonad and between the ventral and dorsal gonads, 6 slides matching the anterior, median and posterior sections of the dorsal and ventral gonads (respectively coded D1, D2, D3 and V1, V2, V3) for 15 individuals were read. The 15 ovaries chosen for this part of the study did not show oocytes with advanced vitellogenesis (vit4), hydrating oocytes (och), or hydrated oocytes (oh). The reading of all 90 slides was done by the 3 operators that had previously validated the inter-calibration exercise. For each section of a single ovary, one of each slide was randomly assigned (cast of dice) to one of the three operators. Each slide was assigned only once, and each operator had a collection of 30 slides to read, composed of one slide from each ovary.

Afterward, the differences (in %) between the minimum and maximum count of each type of cellular structure found throughout all 6 sections, as well as histograms figuring the number of times each cellular structure was counted within the ovaries of a single individual, were established to better visualize the results found for all 15 fish. With the aim of objectively stating the effects of the section position within the gonad, as well as cellular structure occurrences within these sections, general linear models (GLM) were performed.

These models were used to highlight the differences between the 6 slides, for each cellular structure, and for all 15 individuals. The response variable used was the number of times a structure was counted on a single slide divided by the total number of sampled points on that same slide. For the GLM, the error term followed a binomial distribution, and a logit regression model^26,27^ was used. The model results were then analyzed by using the deviances of each variable (the 20 cellular structures). The function drop1^27^ was used to quantify the deviances of each variable by removing them from the whole model alternatively. A principal component analysis (PCA) on the histological structures was established to summarize and plot the reading data.

## Data Availability

For the inter-agent calibration results, as well as the cellular homogeneity verification, a code was set up using RStudio (version 1.2.5001). This code can be accessed by contacting either Carine Sauger (carine.sauger@gmail.com) or Laurent Dubroca (laurent.dubroca@gmail.com).

## References

[1] Alonso-Fernández A, Villegas-Ríos D, Valdés-López M, Olveira-Domínguez B, Saborido-Rey F (2013). Reproductive biology of pollack (*Pollachius pollachius*) from the Galician shelf (north-west Spain). J. Mar. Biol. Assoc. U. K..

[2] Mahé, K., Delpech, J. P. & Carpentier, A. Synthèse bibliographique des principales espèces de Manche orientale et du golfe de Gascogne. *Conv. IFREMER* **2006-0000708**, 78–81 (2007).

[3] FAO. *La situation mondiale des pêches et de l’aquaculture. Atteindre les objectifs de développement durable*. License: CC BY-NC-SA 3.0 IGO (Organisation des Nations Unies pour l’Alimentation et l’Agriculture, 2018).

[4] Brown-Peterson NJ, Wyanski DM, Saborido-Rey F, Macewicz BJ, Lowerre-Barbieri SK (2011). A Standardized Terminology for Describing Reproductive Development in Fishes. Mar. Coast. Fish. Dyn. Manag. Ecosyst. Sci..

[5] ICES. *Report of the Workshop for Advancing Sexual Maturity Staging in Fish (WKASMSF)*. EOSG: No. 38 (International Council for the Exploration of the Sea, 2018).

[6] Gerritsen HD, McGrath D (2006). Variability in the assignment of maturity stages of plaice (*Pleuronectes platessa L*.) and whiting (*Merlangius merlangus L*.) using macroscopic maturity criteria. Fish. Res..

[7] ICES. *Report of the Workshop on sexual maturity staging of cod, whiting, haddock, saithe and hake (WKMSGAD)*. EOSG: No. 57 (International Council for the Exploration of the Sea, 2013).

[8] ICES. *Working Group on Biological Parameters (WGBIOP)*. EOSG: No. 07 (International Council for the Exploration of the Sea, 2018).

[9] ICES. *Report of the Workshop for maturity staging chairs (WKMATCH)*. ACOM: No. 58 (International Council for the Exploration of the Sea, 2012).

[10] Barr WA (1963). The endocrine control of the sexual cycle in the Plaice, *Pleuronectes platessa* (*L*.). I. Cyclical changes in the normal ovary. Gen. Comp. Endocrinol..

[11] Brule T (1987). The reproductive biology and the pathological changes of the plaice *Pleuronectes platessa* (*L*.) after the ‘Amoco Cadiz’ oil spill along the north-west coast of Brittany. J. Mar. Biol. Assoc. U.K..

[12] Lincoln RF (1981). Sexual maturation in female triploid plaice, *Pleuronectes platessa*, and plaice x flounder, *Platichthys flesus*, hybrids. J. Fish Biol..

[13] Miossec L (1984). Altération de l’ovogénèse des plies *Pleuronectes platessa L*. capturées dans les abers Wrac’h et Benoit, depuis la pollution de l’Amoco-Cadiz. Rev. Trav. Inst. Pêch. Marit..

[14] Gabe, M. *Techniques Histologiques*, 2nd edn (Masson, 1968).

[15] Bromley PJ, Ravier C, Witthames PR (2000). The influence of feeding regime on sexual maturation, fecundity and atresia in first-time spawning turbot. J. Fish Biol..

[16] Hoarau G, Rijnsdorp AD, Van Der Veer HW, Stam WT, Olsen JL (2002). Population structure of plaice (*Pleuronectes platessa L*.) in northern europe: microsatellites revealed large-scale spatial and temporal homogeneity. Mol. Ecol..

[17] Aperio Technologies. Aperio image scope (Leica Biosystems Imaging, 2015).

[18] Glagolev AA (1934). Quantitative analysis with the microscope by the ‘point’ method. Miner. Eng. J..

[19] Gundersen HJG, Jensen EB (1987). The efficiency of systematic sampling in stereology and prediction. J. Microsc..

[20] Sauger, C. & Kellner, K. Lexicon of histological structures found in the ovaries and during the oogenesis of the European plaice, *Pleuronectes platessa* (Linné, 1758). *IFREMER* (2019).

[21] Sauger C (2019). Zenodo.

[22] Sauger, C., *et al* Protocol for the determination of histological structures found in the ovaries and during the oogenesis of the European plaice, *Pleuronectes platessa* (Linné, 1758). *IFREMER* (2019).

[23] McHugh ML (2012). Interrater reliability: the kappa statistic. Biochem. Medica.

[24] Hallgren KA (2012). Computing Inter-Rater Reliability for Observational Data: An Overview and Tutorial. Tutor. Quant. Methods Psychol..

[25] Conger AJ (1980). Integration and Generalization of Kappas for Multiple Raters. Psychol. Bull..

[26] Chessel, D. & Thioulouse, J. Modèle linéaire généralisé. *Fiche n°5 d’utilisation du logiciel R*, https://pbil.univ-lyon1.fr/R/pdf/br5.pdf (2013).

[27] Zuur, A. F., Ieno, E. N., Walker, N. J., Saveliev, A. A. & Smith, G. M. *Mixed effects models and extensions in ecology with R, Statistics for biology and health* (Springer, 2009).

